# The In Vitro Anticoccidial Activity of Some Herbal Extracts against *Eimeria* spp. Oocysts Isolated from Piglets

**DOI:** 10.3390/pathogens12020258

**Published:** 2023-02-06

**Authors:** Mihai-Horia Bǎieş, Adriana Györke, Vlad-Dan Cotuţiu, Zsolt Boros, Anamaria Cozma-Petruț, Lorena Filip, Laurian Vlase, Ana-Maria Vlase, Gianina Crişan, Marina Spînu, Vasile Cozma

**Affiliations:** 1 Department of Parasitology and Parasitic Disease, Faculty of Veterinary Medicine, University of Agricultural Sciences and Veterinary Medicine of Cluj-Napoca, 3-5 Mǎnǎştur Street, 400372 Cluj-Napoca, Romania; 2 Department of Bromatology, Hygiene, Nutrition, Faculty of Pharmacy, “Iuliu Haţieganu” University of Medicine and Pharmacy, 6 Pasteur Street, 400349 Cluj-Napoca, Romania; 3 Department of Pharmaceutical Technology and Biopharmaceutics, Faculty of Pharmacy, “Iuliu Haţieganu” University of Medicine and Pharmacy, 12 Ion Creangǎ Street, 400010 Cluj-Napoca, Romania; 4 Department of Pharmaceutical Botany, Faculty of Pharmacy, “Iuliu Haţieganu” University of Medicine and Pharmacy, 23 Gheorghe Marinescu Street, 400337 Cluj-Napoca, Romania; 5 Department of Infectious Diseases, Faculty of Veterinary Medicine, University of Agricultural Sciences and Veterinary Medicine of Cluj-Napoca, 3-5 Mǎnǎştur Street, 400372 Cluj-Napoca, Romania; 6 Academy of Agricultural and Forestry Sciences Gheorghe Ionescu-Siseşti (A.S.A.S.), 61 Mǎrǎşti Bulevard, 011464 Bucharest, Romania

**Keywords:** plant extracts, *Eimeria suis*, *Eimeria debliecki*, oocysts, swine

## Abstract

Coccidiosis in pigs seldom results in important economic losses. However, it can influence growth rates in weaners and it is an important hygiene indicator in swine farms. Certain herbs, along with their extracts, have been used over the course of history in traditional medicine. The aim of this study was to evaluate the in vitro anticoccidial effects of *Allium sativum* L. (garlic), *Artemisia absinthium* L. (wormwood), *Coriandrum sativum* L. (coriander), *Cucurbita pepo* L. (pumpkin), *Satureja hortensis* L. (summer savory), and *Calendula officinalis* L. (marigold) against *Eimeria suis* and *Eimeria debliecki* oocysts. The stock solution of oocysts (58% *E. suis +* 42% *E. debliecki*) was incubated for three days, before adding the tested solutions. The unsporulated *Eimeria* spp. oocysts were then placed in a 3 mL well plate and incubated for 96 h at 27 °C, in a suspension containing serial dilutions of alcoholic plant extracts (5%, 2.5%, 1.25%, 0.625%, and 0.312%). The percentage of sporulated and destroyed oocysts was recorded every 24 h for 96 h. All extracts had a good in vitro anticoccidial effect against oocysts of *Eimeria* spp. compared with the control groups. Oocyst sporulation was significantly (*p* = 0.05) inhibited by the 5% marigold extract (3.6% sporulated oocysts). The same extract had the highest lytic effect on oocysts (65.2% destroyed oocysts). Our results prove that the most effective alcoholic plant extract is the marigold extract, followed, in order of efficiency, by the wormwood, coriander, garlic, pumpkin, and summer savory extracts. This study represents a preliminary contribution for establishing a new generation of natural disinfectants aimed at destroying *Eimeria* oocysts in the context of swine contamination.

## 1. Introduction

Coccidiosis in certain livestock (birds and ruminants), caused by *Eimeria* spp., has a grave economic impact. In pigs, on the other hand, it is considered less significant, as natural infections are only sporadically related to clinical disease [1]. Infections with *Eimeria* spp. are common in pigs worldwide, clinically affecting weaners and fatteners, manifested through diarrhea and weight loss. On the other hand, *Eimeria* infections can serve as an indicator of the overall hygiene within a farm. The lack of productivity, due to subclinical infections, is noticeable when the animals are in a heightened state of production (lactation, gestation). Ruminants and sows, particularly during the periparturient period, displayed an increase in the coproelimination of oocysts [1,2,3]. Globally, the prevalence rates for swine eimeriosis can vary between 5.6 and 77.7%, dependent on age and production types [3,4,5,6]. Resistance to anticoccidial compounds has seen an increase due to the commercial availability of products to the general public, without requiring a veterinarian’s prescription. One major concern is represented by new restrictions on the use of anticoccidial drugs along with the absence of an accessible or scalable anticoccidial vaccine for piglets [7].

Pigs are commonly infected by eight *Eimeria* species including *Eimeria spinose*, *Eimeria porci*, *Eimeria debliecki*, *Eimeria neodebliecki*, *Eimeria perminuta*, *Eimeria polita*, *Eimeria scabra*, and *Eimeria suis*, which can be differentiated by oocyst morphology [8,9]. Infected swine shed thousands of unsporulated oocysts, which within 5–13 days (depending on the *Eimeria* species) and under appropriate environmental conditions (humidity and temperature, as well as oxygen availability) will sporulate and become infectious [8,9]. The wall of *Eimeria* oocyst is a robust structure that is resistant to a variety of physical and chemical threats. Therefore, a critical point to destroy this pathogen is the disturbance of the sporulation process [10].

In swine farms worldwide, cleaning and disinfection are mandatory procedures used as preventative or precautionary measures against bacterial and parasitic infections [11]. Disinfection efficacy was evaluated by either the sporulation inhibition or destruction of the oocysts [12]. Several studies were carried out to assess the effects of physical (ozone, irradiation), chemical (chromium compounds, phenol, ethanol, formaldehyde, etc.), and commercial disinfectants (Preventol, Neopredisan, Dettol, TH4, Virkon^®^S, and others) against the sporulation of *Eimeria* oocysts [12,13,14,15,16,17]. Junior et al. (2007) [12] mention that protozoan oocysts are very resistant to most commercially available disinfectants.

Lastly, an increased interest in safe and effective alternatives aimed at controlling coccidiosis has led to the use of plant extracts, essential oils, and traditional medicinal products, in organic swine farms in particular [18,19,20]. Furthermore, extracts and essential oils from *Artemisia absinthium*, *Allium sativum*, and *Satureja hortensis* were evaluated for their activity on the sporulation of *Eimeria* species, and demonstrated, in vitro, a strong anticoccidial effect [21,22,23]. In organic swine farming, the use of chemicals is prohibited; therefore, plant extracts are a sustainable alternative for controlling parasitic diseases in livestock [24].

The aim of this study was to evaluate the effects of the alcoholic extracts from *Allium sativum* L. (garlic)*, Artemisia absinthium* L. (wormwood)*, Coriandrum sativum* L. (coriander)*, Cucurbita pepo* L. (pumpkin)*, Satureja hortensis* L. (summer savory), and *Calendula officinalis* L. (marigold) on the sporulation of *Eimeria suis* and *Eimeria debliecki* oocysts, isolated from piglets.

## 2. Materials and Methods

### 2.1. Eimeria *spp.* Oocysts Isolation

Oocysts used in this study were isolated from fresh feces of naturally infected piglets, aged between 2 and 4 months, from two free-range farms (Cluj county, Romania). The oocysts were purified by flotation, in a saturated sugar solution, and concentrated as described by Mircean et al. (2011) [25]. The obtained oocysts suspension was mixed with 2.5% potassium dichromate in equal volumes, resulting the stock suspension of oocysts (SOS) [25,26]. The McMaster method was used to establish the number of oocysts/mL. The SOS was preincubated for 3 days at 27 °C, because the sporulation time of *Eimeria* species of swine (5–13 days) differs from other animal species being much longer [8]. The experimental protocol started with the addition of the distilled water, ethanol, and alcoholic plant extracts (APEs).

Furthermore, the *Eimeria* species were identified by the morphology of sporulated oocysts [8]. For this, 5 mL of oocysts suspension was incubated for 14 days, and microphotographs were taken with Olympus DP72 camera (Olympus Corporation, Tokyo, Japan). The morphology and size of 50 oocysts were observed and measured under microscope (Olympus BX61, Olympus Corporation, Tokyo, Japan) using a 400× magnification.

### 2.2. Alcoholic Plant Extracts

The aerial parts of wormwood, marigold, and summer savory, along with coriander fruits, garlic bulbs, and pumpkin seeds, were used. The plant extracts were obtained by adding 10 g of the powdered plants to 100 mL of 70° alcohol (10%). All extractions and chemical analyses were performed at the “Iuliu Hațieganu” University of Medicine and Pharmacy Cluj-Napoca as previously described by Băieș et al. [27]. High-performance liquid chromatography coupled with mass spectrometry (LC/MS) were used for the analysis of major chemical compounds (poliphenols, sterols, tocopherols, sesquiterpene lactones, methoxylated flavones, and sulfoxide). The equipment, techniques, and methods used for analysis of alcoholic plant extracts have already been detailed in a previous publication [27].

### 2.3. Experimental Design

The experiment was performed in 24-well cell culture plates with 5 replicates for each experimental variant. Experimental variants are presented in Table 1. Two controls (ethanol = positive control, and potassium dichromate = negative control) and six APE (AS = *A. sativum*, AA = *A. absinthium*, CS = *C. sativum*, CP = *C. pepo*, CO = *C. officinalis* and SH = *S. hortensis*) were evaluated (Table 1). In each well, 1 mL of stock pre-incubated oocysts suspension (*n* = 15,000) and 1 mL of either APE, ethanol, or distilled water were placed, according to the experimental variant (Table 1). Next, 10% APE (the initial concentration) was tested in five, two-fold serial dilutions as follows: 5% = 50 mg/mL; 2.5% = 25 mg/mL; 1.25% = 12.5 mg/mL; 0.625% = 6.25 mg/mL; and 0.312% = 3.12 mg/mL, which represented the final concentrations of APE. Additionally, ethanol was used in five different concentrations (35%, 17.5%, 8.75%, 4.375%, and 2.187%) that corresponded with the ethanol concentration in the diluted plant extracts. The negative control was performed in five wells [20,22,23].

**Figure 1:**
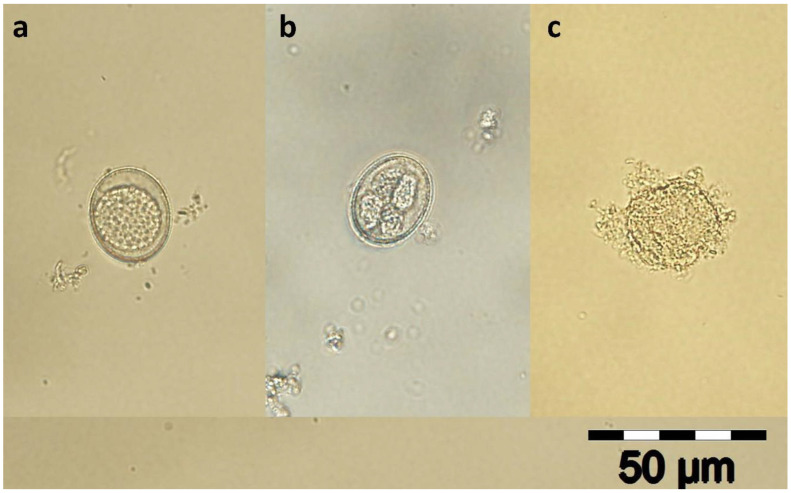
Unsporulated (**a**), sporulated (**b**), and destroyed oocyst of *Eimeria suis* (400×). The concentration of 50 mg/mL of each APE produces complete oocyst wall destruction (**c**).

Afterward, the plates were incubated (Binder incubator BD 260) at 27 °C for 96 h. Every 24 h (4 times in total), one hundred oocysts/well were counted under a light microscope, recording the number of unsporulated, sporulated, and destroyed oocysts (Figure 1). Furthermore, the lethal concentration (LC_50_) for each APE was calculated [28,29].

### 2.4. Statistical Analysis and Ontologies

The mean and standard deviation of the mean were calculated for the number of sporulated and destroyed oocysts at 24, 48, 72, and 96 h. The ANOVA statistic was then used to compare the experimental groups with the control groups, and the experimental groups among themselves. A value of *p* ≤ 0.05 was considered statistically significant. The percentage of destroyed oocysts was adjusted using Abbott’s formula (destroyed oocysts in experimental variant − destroyed oocysts in corresponding ethanol variant)/(100 − destroyed oocysts in corresponding ethanol variant) × 100 [30]. The lethal concentration LC_50_ was calculated using the Microsoft Excel program.

The ontologies/pathogens, diseases, medicinal plants, and chemical compounds used in the experiment were described in Table S1, in accordance with the PPILOW project data management plan.

## 3. Results

### 3.1. Analysis of Plant Extracts

The major biological compounds identified by biochemical analysis of the 10% alcoholic plant extracts were as follows: poliphenols and sterols for *A. absinthium*; poliphenols, tocopherols, and sulfoxide for *A. sativum*; tocopherols and sterols for *C. pepo*; poliphenols, tocopherols, sterols, methoxylated flavones, and sesquiterpene lactones for *C. sativum*; poliphenols, tocopherols, and sterols for *C. officinalis;* and poliphenols, tocopherols, sterols, and methoxylated flavones for *S. hortensis*. These compounds were presented in detail in the supplementary material in Table S2, the data being previously published by Băieș et al. [27].

### 3.2. In Vitro Antiparasitic Activity of APE against Eimeria *spp.* Oocysts

The stock oocysts suspension contained *Eimeria debliecki* (42%) and *Eimeria suis* (58%) identified by their morphology.

*Eimeria debliecki* oocysts are of ovoid or ellipsoid shape, lacking micropyle and residuum, with a smooth, colorless, or lightly yellow wall. The dimension of these oocysts was 19.2 (16.2–23.9) × 14.1 (11.7–17.6) μm. The sporocysts measured 11.5 (6.9–16.1) × 5.1 (3.7–7.3) μm with the presence of a sporocyst residuum. Sporulation time varied between 5 and 7 days.

*Eimeria suis* oocysts are ellipsoidal without micropyle and oocyst residuum. Their size was 18.1 (15.6–22.7) × 13.8 (12.5–17.7) μm. The oocysts wall resembled that of *E. debliecki*, while sporocysts were generally shorter and stumpy, measuring 8.6 (6.8–10.9) × 5.9 (4.5–7.1) μm. Sporulation time was about 5–6 days.

The starting time of the experimental protocol was considered when the distilled water, ethanol, and APE were added, recording the results from this point onward. The percentages of sporulated and destroyed oocysts were calculated for control and experimental groups at 24, 48, 72, and 96 h (Tables S3 and S4).

**Figure 2:**
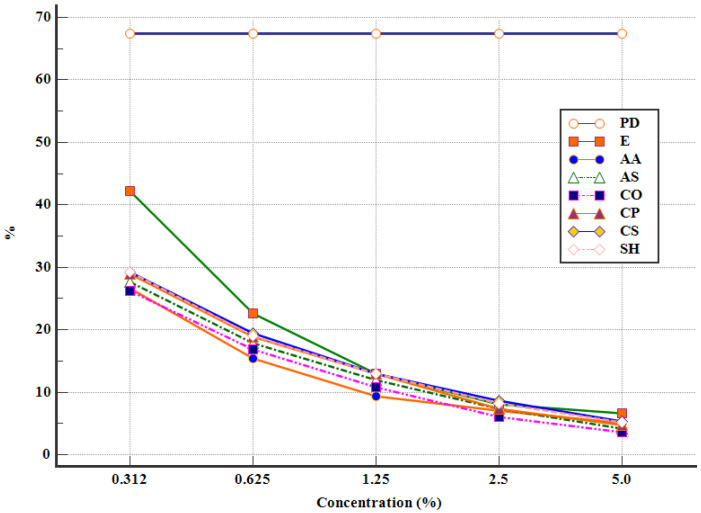
Sporulated oocysts after 96 h of incubation (PD—potassium dichromate, E—ethanol, AA—*A. absinthium*, AS—*A. sativum*, CO—*C. officinalis*, CP—*C. pepo*, CS—*C. sativum*, SH—*S. hortensis*).

The sporulation process began after 48 h of incubation, in all experimental variants. The lowest percentages of sporulated oocysts were recorded for CO 5 (3.6 ± 0.3%), AS 5 (4.2 ± 0.75%), and AA 5 (5.0 ± 0.79%), after 96 h of incubation. The concentration of the plant extract was inversely proportionate with the percentage of sporulated oocysts, in all of the studied plants (Figure 2).

**Figure 3:**
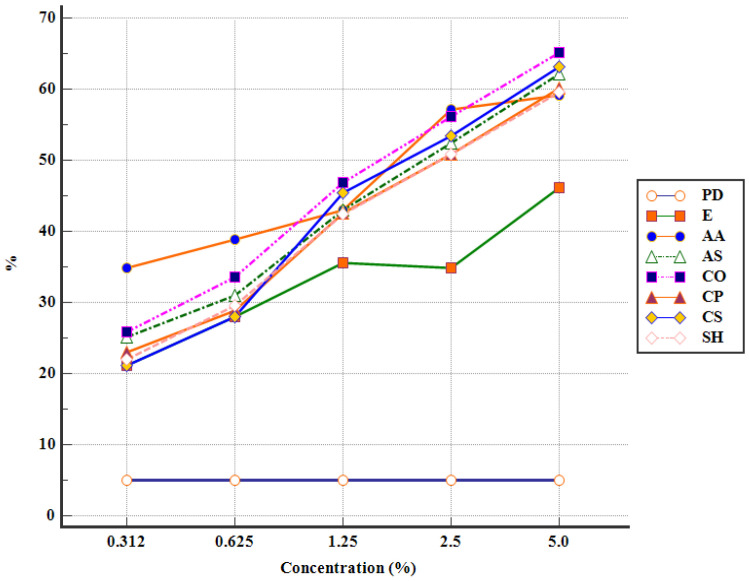
Destroyed oocysts after 96 h of incubation (PD—potassium dichromate, E—ethanol, AA—*A. absinthium*, AS—*A. sativum*, CO—*C. officinalis*, CP—*C. pepo*, CS—*C. sativum*, SH—*S. hortensis*).

The efficacy of the APEs increased, following 72 h of incubation, resulting in a high number of destroyed oocysts for AA 2.5 (32.12 ± 3.81%), CO 2.5 (29.72 ± 3.14%), and CO 5 (27.5 ± 2.18%) (Table 2). Furthermore, after 96 h of incubation, the highest percentage of destroyed oocysts was noticed in CO 5 (35.01 ± 1.93%), followed by AA 2.5 (33.82 ± 4.87%) and CO 2.5 (32.51 ± 1.72%) (Table 2). Similar results were obtained with CS 5 (31.27 ± 4.42%), AS 5 (29.38 ± 3.16%), and CS 2.5 (28.08 ± 3.29%) (Table 2). On the other hand, the lowest percentage of destroyed oocysts, after 96 h of incubation, was observed in CP 0.625 (0.8 ± 0.12%), SH 0.312 (0.95 ± 0.2%), and SH 0.625 (1.89 ± 0.3%) (Table 2). A general and summarized outlook on the efficacy of APEs after 96 h of incubation can be observed in Figure 2 and Figure 3.

The lethal concentration (LC_50_) of each APE was presented in Table 3. For the first two days of incubation, LC_50_ was not calculated_,_ since none of the APEs managed to destroy at least 50% of the *Eimeria* spp. oocysts. After 72 h of incubation, the lowest lethal concentration of APE was recorded by *C. officinalis* (LC_50_ = 24.55 mg/mL) followed, in order, by *C. sativum*, *A. sativum*, *A. absinthium*, *C. pepo,* and *S. hortensis* (Table 3). Furthermore, after 96 h of incubation, *C. officinalis* (LC_50_ = 16.98 mg/mL) induced the lowest LC_50_ value, followed by *A. absinthium*, *C. sativum*, *A. sativum*, *C. pepo,* and *S. hortensis* (Table 3).

All the APEs affected the sporulation of *Eimeria* spp. oocysts, as well as their integrity, in a dose-dependent manner. The marigold alcoholic extract, at a concentration of 50 mg/mL (5%), was the most effective of the APEs.

The results are statistically significant (*p* ≤ 0.05) between ethanol and APEs, for all studied plants, depending on the concentration of the extract (Tables S3 and S4). Statistically relevant values were also observed between the experimental groups at different concentrations, with the anticoccidial efficiency as a variable (Table S3, Table S4 and Table 2). We performed the ponderation both with potassium dichromate and with ethanol, the final results representing the real effectiveness of the APEs (Table 2).

## 4. Discussion

There is a constant and inherent requirement in finding alternative options for disinfectants, with an antiparasitic effect in livestock. The overall aim is to use molecules that are deemed safe for animals, with little to no spillover effect on the environment. The current trend involves using products based on natural compounds that have a proven antiparasitic activity, with very few side effects. Although swine eimeriosis is of low pathogenicity compared to isosporosis, our results can be extrapolated to other species where the disease is more clinically relevant (birds, rabbits, ruminants), and possibly even in piglet isosporosis.

This study evaluated the in vitro anticoccidial activity of the alcoholic extract of garlic, wormwood, coriander, pumpkin, marigold, and summer savory on the sporulation of oocysts of *Eimeria suis* and *Eimeria debliecki*. In swine, the inhibition of the sporulation process is a common criterion for evaluating anticoccidial properties [31]. Therefore, the method used in this study is cost-effective and time-efficient. Due to a lack of studies on *Eimeria* oocysts in pigs, we have focused our attention, in large part, on existing reports on broiler chicken coccidiosis. Phytochemicals may possess an anti-sporulation property by interfering with the physiological process necessary for sporulation, namely, preventing the access of oxygen and thus inhibiting the enzyme responsible for sporulation [19]. Biological compounds such as polyphenols, tocopherols, flavonoids, sesquiterpene lactones, and sulfoxide have demonstrated strong anticoccidial properties, both in vitro and in vivo, and can, therefore, be used as substitutes for commercial disinfectants [32,33,34,35].

Garlic is rich in organosulfur compounds (e.g., allicin, diallyl sulfide, and diallyl trisulfide), which are key components due to their antiparasitic effects [36]. Of these, allicin has exhibited a clear effect on the development of *Eimeria tenella* sporozoites in cell cultures with almost complete inhibition at a dose of 1.8 mg/mL [21]. Allicin also has a strong antiparasitic effect on other protozoa such as *Giardia* spp., *Leishmania* spp., *Trichomonas vaginalis*, etc. [37,38]. In another study performed on cell cultures, the garlic essential oil reached a maximum inhibition of 70% on *E. tenella* sporozoites after 24 h at a concentration of 50 μg/mL [18]. Gadelhaq et al. (2018) [20] concluded that garlic powder displayed no effect on oocysts sporulation, and the action is attributed to the toughness of the oocyst wall, acting as a stumbling block. All of the aforementioned criteria underlined the necessity for an investigation into the garlics’ ability to inhibit sporulation, as well as to destroy *Eimeria* oocysts. A different study reported the inhibitory effect of aqueous garlic extract on sporulation through the supposed anticoccidial effect of the organosulfur compound [39]. In the current study, garlic demonstrated a good activity even at the minimum concentration of 3.12 mg/mL in both inhibiting sporulation and destroying oocysts. The garlic extract demonstrated a strong efficacy at all tested concentrations, an effect comparable with *C. officinalis*, *A. absinthium*, and *C. sativum*. This effect can mainly be attributed to sulfoxide and polyphenols, which are in a fairly high concentration in this plant.

The main bioactive components of wormwood are artemisinin and quercetin [40]. Numerous experimental studies have been performed on the efficacy of *A. absinthium* and *Artemisia annua* in coccidiosis with positive results on the inhibitory effect on the sporulation of *Eimeria* spp. [22,41]. Reports also suggest that artemisinin could be effective against toxoplasmosis, giardiasis, isosporosis, babesiosis, and leismaniosis [37]. The in vitro antiparasitic effect of the wormwood alcoholic extract and essential oil against *Eimeria* oocysts has been demonstrated, with a lethal concentration of under 1 mg/mL [19,23]. In the present study, the wormwood ethanolic extract is the second most effective extract, at all concentrations used. The anticoccidial effect is probably due to the polyphenols found in abundance in this plant. Although the mechanism of these compounds is poorly understood, their inhibitory effect on embryogenesis is high. The minimum lethal concentration of the ethanolic extract of *A. absinthium* was 3.12 mg/mL.

Coriander is an aromatic plant with medicinal attributes and antiparasitic activity. The phytochemical screening of *C. sativum* showed that it contained essential oils, flavonoids, fatty acids, isocoumarin sterols, and phenolic compounds [42,43,44]. Coriander dried fruits contain about 1% essential oil, with linalool as the main active component [43]. A herbal formula containing *C. sativum*, among other plants, was successfully used as a natural anticoccidial molecule against eimeriosis [45]. Coriander essential oil demonstrated significant antiprotozoal effects against *Leismania amazonensis* and *Leismania infantum* amastigotes and promastigotes [46,47]. Aqueous and alcoholic coriander extracts have a weak in vitro effect against *Cryptosporidium* [48]. Boros et al., 2021, [49] showed that *C. sativum* ethanolic extracts completely inhibited the mobility of *Trichinella spiralis* and *Trichinella britovi* larvae. In vitro, coriander essential oil exhibited a strong anthelmintic efficacy against ovine gastrointestinal nematodes by inhibiting egg hatching, larval development, and motility [50,51]. In the present study, the coriander extract had the third highest effectiveness in inhibiting sporulation and destroying oocysts due to its rich polyphenol concentration. It must, however, be noted that their concentration in *C. sativum* is lower compared to that found in wormwood.

Pumpkin seeds contain several active constituents: essential fatty acids, amino acids, phytosterols (β-sitosterol), minerals, vitamins, and cucurbitin. The latter is the main chemically active compound in pumpkin seeds responsible for its antiparasitic effect [52,53]. Elhadi et al. (2013) [54] proved that cucurbitacin E and cucurbitacin L have a strong efficacy against *Giardia lamblia*, in vitro, at a concentration of 5 μg/mL after 5 days. Pumpkin is known for its antihelmintic properties, and while studies on the effect against protozoa are few, they show a strong activity against *Plasmodium* spp. [30]. Salman et al. (2022) [55] reported that pumpkin seeds, at concentrations of 200 μg/mL, displayed a significant inhibition of the number of *Blastocystis* (the most common protozoa in humans) throughout the incubation period, and the concentration of 400 μg/mL had the strongest antiparasitic effect. In an in vitro study, it was noted that the concentration of 5 and 10 mg/mL of ethanolic extract in pumpkin seeds had a complete inhibitory effect on the growth of *Histomonas meleagridis*, *Tetratrichomonas gallinarum*, and *Blastocystis* spp., after 48 h of incubation [56]. In the present study, the ethanolic pumpkin seed extract demonstrated a weak activity on inhibiting the sporulation of *Eimeria* oocysts at all studied concentrations (3.125–50 mg/mL), possibly due to the lack of flavonoid, polyphenols, and sesquiterpene lactones which are known for their anticoccidial effect.

Summer savory essential oil contains p-cymene, γ-terpinene, carvacrol, and thymol. Its disinfectant property is attributed to thymol [57]. Felici et al. (2020) [58] demonstrated the antiparasitic effect of saponins, thymol, and carvacrol on *Eimeria* spp. sporozoites in cell cultures. Additionally, carvacrol and thymol produced the destruction of *Eimeria* oocysts [23]. Thyme, belonging to the same family as summer savory, exhibited a strong anticoccidial effect in vitro [59]. In the current study, the *S. hortensis* ethanolic extract both destroyed and inhibited the development of oocysts, at all tested concentrations. Although the plants’ content is rich in polyphenols and methoxylated flavones, this extract had the lowest efficacy in inhibiting the sporulation as well as destroying oocysts, results comparable to the control group treated with ethanol.

*C. officinalis* is a medicinal plant rich in carotenoids, coumarins, volatile oils, flavonoids, saponins, sterols, and phenolic acids, which are known as biologically active compounds with multiple applications in phytotherapy [60,61]. Nikmehr et al. (2014) [62] demonstrated that marigold methanolic extract inhibited the growth of *Leismania major* promastigotes and amastigotes, while also reducing the number of macrophage amastigotes. The antihelmintic activity of certain bioactive principles (triterpenoid saponins, glycosides of oleanic acid) isolated from marigold, against free-living stages of *Heligmosomoides bakery* and *Heligmosomoides polygyrus* was already reported on [63,64]. The essential oil of *C. officinalis* killed L1-2 larvae of *Strongiloides papillosus* but had no effect on the embryogenesis of *A. suum* eggs [65]. In the present study, the ethanolic marigold extract displayed the strongest anticoccidial activity, compared to the other plants studied. The effectiveness of *C. officinalis* is probably attributed to the high concentration in poliphenols and tocopherols.

The unsporulated oocyst is the environmentally resistant form of *Eimeria* spp., while their sporulated forms represent the infective stage of the parasite. Despite the high anticoccidial activity of ethanol, we considered that the activity of various alcoholic plant extracts (APEs) can still be objectively quantified. Ethanol was used as a control in order to correctly assess the effectiveness of the APEs. Gadelhaq et al. (2018) [25] reported that 70% ethanol completely inhibited the sporulation of *Eimeria* oocysts after 48 h of incubation. Moreover, we also wanted to assess the antiprotozoal effect of APEs, using different concentrations, while also entertaining the possibility of using them as a disinfectant. Ethanol alone has a disinfectant activity, yet, used in combination with plants it potentiates their effects, which offers added value to plant extracts. The organic requirements restrict the use of chemicals. Therefore, natural plant products may represent an effective solution for pathogen control in organic swine farming [66]. In the present study, the stock solution of oocysts was previously incubated for three days to reduce, as much as possible, the contact time between the oocysts and the tested solutions; thus, the disinfecting action of the plant extracts is similar to that used in field conditions.

The screening performed on the six alcoholic plant extracts tested confirms the antiparasitic activity (the highest percentage of destroyed oocysts as well as the lowest percentage of sporulated oocysts) following contact with all APEs used in a dose-dependent manner, at a concentration ranging between 3.125 and 50 mg/mL. Our results prove that the most effective APE is marigold followed, in order, by wormwood, coriander, garlic, pumpkin, and summer savory. This in vitro study concludes that all alcoholic plant extracts induced a pronounced anticoccidial effect, which in turn is directly proportionate to the concentration of the plant extract.

## 5. Conclusions

This study is one of the few performed on *Eimeria* spp. oocysts isolated from piglets. Statistical analysis showed that all plant extracts were effective in inhibiting the sporulation of both *E. suis* and *E. debliecki* oocysts as well as destroying them, while a minor, statistically non-significant percentage of oocysts remained sporulated. The alcoholic extracts of *C. officinalis*, *A. absinthium*, and *C. sativum* were the most potent and obtained the lowest LC_50_ values.

As our in vitro results demonstrated that the APEs at higher concentrations had a dual effect, both inhibitory and destructive, their use as disinfectants in livestock shelters seems encouraging. In order to obtain the strongest anticoccidial effect, the implementation of herbal formulas which contain the most effective alcoholic plant extracts is needed.

Further investigation on the isolation, purification, toxicity, and mechanism of action of the aforementioned major compounds (of tested plants) is required.

## Figures and Tables

**Table 1:** Experimental design.

Groups	Concentration (%)	Abbreviations	Content/Well
**Potassium** **dichromate**	0.625	PD	1 mL SOS (0.5 mL OS + 0.5 mL 2.5% PD) + 1 mL DW
**Ethanol**	35	E 35	1 mL SOS + 1 mL 70% E
	17.5	E 17.5	1 mL SOS + 1 mL 35% E
	8.75	E 8.75	1 mL SOS + 1 mL 17.5% E
	4.375	E 4.375	1 mL SOS + 1 mL 8.75% E
	2.187	E 2.187	1 mL SOS + 1 mL 4.37% E
**Alcoholic plant extracts**	5	AS 5, AA 5, CS 5, CP 5, SH 5, CO 5	1 mL SOS + 1 mL 10% APE
	2.5	AS 2.5, AA 2.5, CS 2.5, CP 2.5, SH 2.5, CO 2.5	1 mL SOS + 1 mL 5% APE
	1.25	AS 1.25, AA 1.25, CS 1.25, CP 1.25, SH 1.25, CO 1.25	1 mL SOS + 1 mL 2.5% APE
	0.625	AS 0.625, AA 0.625, CS 0.625, CP 0.625, SH 0.625 CO 0.625	1 mL SOS + 1 mL 1.25% APE
	0.312	AS 0.312, AA 0.312, CS 0.312, CP 0.312, SH 0.312, CO 0.312	1 mL SOS + 1 mL 0.625% APE

OS—oocysts suspension, SOS—stock oocysts suspension (oocysts suspension mixed with potassium dichromate in equal volumes), PD—potassium dichromate, DW—distilled water, E—ethanol, APEs—alcoholic plant extract, AS—*A. sativum*, AA—*A. absinthium*, CP—*C. pepo*, CS—*C. sativum*, SH—*S. hortensis*, CO—*C. officinalis*.

**Table 2:** The percentage of destroyed oocysts (mean ± SDM) from the experimental groups using Abbot formula.

Time (Hours)	AS 5	AA 5	CS 5	CP 5	SH 5	CO 5
**24**	16.44 ± 5.22 ^a^	9.23 ± 3.05 ^a^	18.5 ± 3.59 ^a^	15.6 ± 2.88 ^a^	15.03 ± 2.72 ^a^	18.94 ± 1.93 ^a^
**48**	16.12 ± 6.38 ^a^	6.98 ± 2.04 ^a^	17.74 ± 3.34 ^a^	13.05 ± 2.29 ^a^	14.87 ± 2.54 ^a^	18.06 ± 2.77 ^a^
**72**	23.78 ± 5.47 ^a^	13.2 ± 3.09 ^a^	23.97 ± 3.74 ^a^	21.97 ± 2.98 ^a^	20.36 ± 2.88 ^a^	27.5 ± 2.18 ^a^
**96**	29.38 ± 3.16 ^ab^	23.96 ± 3.71 ^ab^	31.27 ± 4.42 ^ab^	25.36 ± 2.38 ^ab^	24.21 ± 2.45 ^b^	35.01 ± 1.93 ^a^
	**AS 2.5**	**AA 2.5**	**CS 2.5**	**CP 2.5**	**SH 2.5**	**CO 2.5**
**24**	15.94 ± 4.66 ^a^	16.03 ± 4.82 ^a^	18.96 ± 3.68 ^a^	15.2 ± 2.92 ^a^	14.1 ± 2.54 ^a^	18.68 ± 2.07 ^a^
**48**	14.72 ± 4.47 ^a^	23.46 ± 4.79 ^a^	18.78 ± 3.49 ^a^	12.47 ± 2.94 ^a^	13.03 ± 2.17 ^a^	17.8 ± 2.55 ^a^
**72**	25.79 ± 6.68 ^a^	32.12 ± 3.81 ^a^	25.6 ± 3.24 ^a^	22.07 ± 2.82 ^a^	22.64 ± 3.27 ^a^	29.72 ± 3.14 ^a^
**96**	26.45 ± 6.66 ^a^	33.82 ± 4.87 ^a^	28.08 ± 3.29 ^a^	24.02 ± 3.28 ^a^	24.15 ± 2.79 ^a^	32.51 ± 1.72 ^a^
	**AS 1.25**	**AA 1.25**	**CS 1.25**	**CP 1.25**	**SH 1.25**	**CO 1.25**
**24**	6.22 ± 3.28 ^a^	9.44 ± 3.39 ^a^	9.6 ± 2.29 ^a^	4.99 ± 1.34 ^a^	4.49 ± 1.02 ^a^	8.5 ± 1.51 ^a^
**48**	5.21 ± 3.21 ^a^	6.85 ± 2.54 ^a^	8.39 ± 2.7 ^a^	3.97 ± 1.12 ^a^	3.72 ± 0.99 ^a^	8.39 ± 1.81 ^a^
**72**	11.05 ± 3.17 ^ab^	11.14 ± 2.93 ^ab^	11.65 ± 2.23 ^ab^	7.42 ± 1.83 ^ab^	5.97 ± 1.19 ^b^	14.18 ± 1.94 ^a^
**96**	10.46 ± 4.28 ^a^	10.41 ± 2.3 ^a^	14.31 ± 2.52 ^a^	10.23 ± 1.73 ^a^	9.93 ± 1.88 ^a^	16.31 ± 1.76 ^a^
	**AS 0.625**	**AA 0.625**	**CS 0.625**	**CP 0.625**	**SH 0.625**	**CO 0.625**
**24**	4.77 ± 2.32 ^a^	8.3 ± 3.8 ^a^	8.84 ± 2.32 ^a^	3.73 ± 1 ^a^	3.21 ± 0.8 ^a^	7.6 ± 1.41 ^a^
**48**	3.33 ± 1.96 ^a^	12.97 ± 3.24 ^a^	10.43 ± 2.78 ^a^	4.31 ± 1.29 ^a^	3.81 ± 1.05 ^a^	9.74 ± 1.87 ^a^
**72**	4.14 ± 2.22 ^ac^	14.17 ± 3.05 ^a^	5.57 ± 1.88 ^ac^	1.34 ± 0.46 ^c^	3.9 ± 0.94 ^bc^	8.71 ± 1.58 ^ab^
**96**	3.82 ± 2.17 ^bc^	14.81 ± 2.99 ^a^	5.94 ± 1.36 ^bc^	0.8 ± 0.12 ^c^	1.89 ± 0.3 ^c^	7.49 ± 1.34 ^b^
	**AS 0.312**	**AA 0.312**	**CS 0.312**	**CP 0.312**	**SH 0.312**	**CO 0.312**
**24**	2.4 ± 1.48 ^ab^	11.01 ± 4.16 ^a^	5.22 ± 1.89 ^ab^	1.24 ± 0.42 ^b^	1.47 ± 0.38 ^b^	4.75 ± 1.16 ^ab^
**48**	3.58 ± 2.07 ^b^	14.89 ± 3.44 ^a^	6.57 ± 1.54 ^b^	1.85 ± 0.51 ^b^	2.1 ± 0.62 ^b^	6.55 ± 1.32 ^b^
**72**	3.31 ± 2.04 ^bc^	14.92 ± 3.69 ^a^	1.89 ± 0.65 ^bc^	0.54 ± 0.12 ^c^	0.5 ± 0.09 ^c^	6.57 ± 1.31 ^b^
**96**	5.06 ± 3.22 ^bc^	17.26 ± 3.09 ^a^	1.9 ± 0.65 ^bc^	2.06 ± 0.58 ^bc^	0.95 ± 0.2 ^c^	5.74 ± 1 ^b^

SDM—standard deviation of mean, AS (*A. sativum*), AA (*A. absinthium*), CP (*C. pepo*), CS (*C. sativum*), SH (*S. hortensis*), CO (*C. officinalis*). Values with no common superscript in a column within an experiment were significantly different (*p* ≤ 0.05).

**Table 3:** The LC_50_ of each APE after 72 and 96 h of incubation.

Time (Hours)	AS (mg/mL)	AA (mg/mL)	CS (mg/mL)	CP (mg/mL)	SH (mg/mL)	CO (mg/mL)
**72**	28.84	31.62	28.18	33.11	35.48	24.55
**96**	21.88	18.62	20.42	23.44	23.99	16.98

AS (*A. sativum*), AA (*A. absinthium*), CP (*C. pepo*), CS (*C. sativum*), SH (*S. hortensis*), CO (*C. officinalis*).

## Data Availability

Not applicable.

## Supplementary Materials

The following supporting information can be downloaded at: https://www.mdpi.com/article/10.3390/pathogens12020258/s1, Table S1: Ontologies/pathogens, diseases, medicinal plants, and chemical compounds used in experiment; Table S2: The LC/MS analysis of chemical compounds in alcoholic plant extracts (10%); Table S3: The percentage of sporulated oocysts (mean ± SDM) from the experimental groups and controls; Table S4: The percentage of destroyed oocysts (mean ± SDM) from the experimental groups and controls.
