# Supplementary material for: The In Vitro Anticoccidial Activity of Some Herbal Extracts against Eimeria spp. Oocysts Isolated from Piglets
Source: Pathogens. 2023 Feb 6;12(2):258. doi: 10.3390/pathogens12020258 (PMC9964294; doi:10.3390/pathogens12020258)
Supplement: Supplementary file 1 [file pathogens-12-00258-s001.zip › pathogens-2087172-supplementary.pdf]

Supplementary data

Table S1. Ontologies/pathogens, diseases, medicinal plants and chemical compounds used in experiment.

**Traits**	**ATOL***, **AHOL****, **OPL*****, **IPNI******, and **ChEBI******* References
**Parasite load traits**	
Parasite Oocysts (OPG)	Oocyst Stage
**Parasite used**	
Eimeria spp.	AHOL_0004070
**Disease description**	
Coccidiosis	AHOL_0005374
**Medicinal plants used**	
Artemisia absinthium L.	300106-2
Allium sativum L.	528796-1
Coriandrum sativum L.	840760-1
Calendula officinalis L	187894-1
Cucurbita pepo L.	292416-1
Satureja hortensis L.	457680-1
**Chemical compounds detected**	
Polyphenols (μg/mL)	26195
Sterols (μg/mL)	15889
Tocopherols (ng/mL	135821
Sesquiterpene lactones (ng/ml)	37667
Methoxylated flavones (ng/mL)	25241
Sulfoxide (μg/mL)	22093

*Traits in reference to the ontology ATOL: https://www.atol-o.com/en/atol-2/; **Traits in reference to the ontology AHOL: https://www.atol-onntologytology.com/ahol/; *** Ontology for Parasite Life cycle: http://wiki.aiisc.ai/index.php/Ontology_for_Parasite_Life_Cycle; **** International Plant Names Index-IPNI : https://www.ipni.org/p/3; ***** Chemical Entities of Biological Interest-ChEBI: https://www.ebi.ac.uk/chebi/.

Table S2. The LC/MS analysis of chemical compounds in alcoholic plant extracts (10%) (Băieș et al., 2022) [27])

Chemical class	Chemical compound	Plant species and plant part used for extract preparation and the results of LC-MS analysis					
		***Allium sativum* L.**	***Artemisia absinthium* L.**	***Coriandrum sativum* L.**	***Cucurbita pepo* L.**	***Calendula officinalis* L.**	***Satureja hortensis* L.**
		bulb	aerial part	fruit	seed	aerial part	aerial part
**Polyphenols (μg/mL)**	Chlorogenic acid	-	107.15	4.177	-	220.767	<LOQ
	Caffeic acid	1.221	-	-	-	-	<LOQ
	p-coumaric acid	-	0.621	0.501	-	-	1.464
	Ferulic acid	0.456	0.759	0.759	-	-	0.557
	Sinapic acid	0.228	-	-	-	-	-
	Vitexin	-	1.631	-	-	-	-
	Isoquercitrin	-	56.754	-	-	38.877	6.515
	Rutoside	-	3.826	<LOQ	-	18.819	<LOQ
	Quercitrin	-	1.113	-	-	<LOQ	0.365
	Quercetol	-	6.285	-	-	-	0.394
	Luteolin	-	1.159	-	-	-	6.621
	Kaempferol	-	3.666	-	-	-	-
	Apigenin	-	0.481	-	-	-	2.442
	Syringic acid	-	1.85	0.09	-	1.51	2.28
	Protocatechuic acid	-	1.32	-	-	0.67	0.95
	Vanillic acid	-	1.98	0.94	-	0.44	0.65
**Tocopherols (ng/mL)**	α-tocopherol	36.1	50.0	-	-	61.6	86.8
	γ-tocopherol	-	23.8	-	446.0	248.9	89.0
	Δ-tocopherol	-	5.0	-	23.2	9.3	13.2
**Sterols (μg/mL)**	Ergosterol	-	0.344	0.584	-	0.500	1.420
	Stigmasterol	-	34.831	9.675	22.024	72.888	14.215
	Β-sitosterol	-	140.985	31.548	5.355	241.997	313.315
	Campesterol	-	3.329	1.780	0.358	1.635	6.140
**Methoxylated flavones (ng/mL)**	Jaceosidin	-	-	-	-	-	8820.76
	Hispidulin	-	3047.92	-	-	-	2483.00
	Eupatorin	-	976.53	-	-	-	-
	Casticin	-	15384.14	-	-	-	-
	Acacetin	-	-	-	-	-	12691.97
**Sesquiterpene lactones (ng/ml)**	α-santonin	-	450.52	-	-	-	-
	Vulgarin	-	6499.39	-	-		-
**Sulfoxide (μg/mL)**	Aliin	14.726	-	-	-	-	-

LC/MS—high performance liquid chromatography coupled with mass spectrometry; "-"—Not found; <LOQ—identified based on MS spectra but not determined quantitatively, below limit of quantification.

Table S3. The percentage of sporulated oocysts (mean ±SDM) from the experimental groups and controls.

Time (hours)	PD	E 35	AS 5	AA 5	CS 5	CP 5	SH 5	CO 5
**72**	57.6 ± 2.97^{a}	3.8 ± 0.92^{b}	2.6 ± 0.77^{b}	2.6 ± 0.76^{b}	4 ± 0.84^{b}	2.6 ± 0.62^{b}	3.4 ± 0.57^{b}	1.6 ± 0.19^{b}
**96**	67.4 ± 5.22^{a}	6.6 ± 0.81^{b}	4.2 ± 0.75^{bc}	5 ± 0.79^{bc}	5.4 ± 0.68^{bc}	4.8 ± 0.45^{bc}	5.2 ± 0.32^{bc}	3.6 ± 0.3^{c}
	**PD**	**E 17.5**	**AS 2.5**	**AA 2.5**	**CS 2.5**	**CP 2.5**	**SH 2.5**	**CO 2.5**
**72**	57.6 ± 2.97^{a}	5.4 ± 1.63^{b}	5 ± 0.91^{b}	4.8 ± 0.82^{b}	6.8 ± 0.66^{b}	6.2 ± 0.89^{b}	5.8 ± 0.32^{b}	4.2 ± 0.46^{b}
**96**	67.4 ± 5.22^{a}	8 ± 0.71^{b}	7.4 ± 0.66^{b}	7 ± 0.61^{b}	8.6 ± 0.81^{b}	7.4 ± 0.47^{b}	8.2 ± 0.32^{b}	6 ± 0.83^{b}
	**PD**	**E 8.75**	**AS 1.25**	**AA 1.25**	**CS 1.25**	**CP 1.25**	**SH 1.25**	**CO 1.25**
**72**	57.6 ± 2.97^{a}	9.6 ± 2.67^{b}	8.6 ± 0.88^{b}	7.4 ± 1.15^{b}	10.4 ±0.81^{b}	9.4 ± 0.74^{b}	9.8 ± 0.49^{b}	7.8 ± 0.3^{b}
**96**	67.4 ± 5.22^{a}	13 ± 2.18^{bc}	12 ± 1.08^{bc}	9.4 ± 1.15^{c}	13 ± 0.71^{bc}	13 ± 0.73^{bc}	15 ± 0.61^{b}	10.8 ±0.46^{c}
	**PD**	**E 4.375**	**AS 0.625**	**AA 0.625**	**CS 0.625**	**CP 0.625**	**SH 0.625**	**CO 0.625**
**72**	57.6 ± 2.97^{a}	19.4 ±1.91^{b}	15.2 ± 1.5^{bc}	12.4 ± 1.6^{c}	16.4±0.51^{bc}	16.2 ± 0.78^{bc}	16 ± 0.6^{bc}	13.8±0.92^{bc}
**96**	67.4 ± 5.22^{a}	22.6 ±1.29^{b}	17.8 ± 2.78^{b}	15.4 ±1.15^{b}	19.4 ±1.21^{b}	18.8 ± 1.42^{b}	19 ± 1.19^{b}	16.8 ±1.38^{b}
	**PD**	**E 2.187**	**AS 0.312**	**AA 0.312**	**CS 0.312**	**CP 0.312**	**SH 0.312**	**CO 0.312**
**72**	57.6 ± 2.97^{a}	36.4 ±2.21^{b}	22.8 ± 2.14^{c}	22.7 ±1.75^{c}	24.8 ±0.66^{c}	23.8 ± 1.2^{c}	24.2 ±1.35^{c}	21.6 ±1.02^{c}
**96**	67.4 ± 5.22^{a}	42.2 ±1.83^{b}	27.6 ± 2.26^{c}	26.6 ±1.35^{c}	29.2 ±0.86^{c}	29 ± 1.04^{c}	29.3 ±0.73^{c}	26.2 ± 1.1^{c}

SDM-standard deviation of mean, E (ethanol), PD (potassium dichromate), AS (*A. sativum*), AA (*A. absinthium*), CP (*C. pepo*), CS (*C. sativum*), SH (*S. hortensis*), CO (*C. officinalis*). Values with no common superscript in a column within an experiment were significantly different ($p \leq 0.05$).

Table S4. The percentage of destroyed oocysts (mean ±SDM) from the experimental groups and controls.

Time (hours)	PD	E 35	AS 5	AA 5	CS 5	CP 5	SH 5	CO 5
**24**	6 ± 3.39^{d}	30.2 ± 2.31^{c}	41.8 ±2.29^{ab}	36.8 ±1.85^{bc}	43.2 ±1.77^{ab}	41.2 ±1.3^{ab}	40.8 ±1.05^{ab}	43.6 ±1.13^{a}
**48**	5.8 ± 1.3^{c}	38.4 ± 2.58^{b}	48.6 ±2.96^{ab}	43 ±2.98^{ab}	49.6±1.86^{ab}	46.6 ±1.25^{ab}	47.8 ±1.45^{ab}	49.8 ±1.45^{a}
**72**	5.8 ±1.48^{d}	43 ± 3.2^{c}	56.6 ±2.9^{ab}	50.6 ±2.8^{bc}	56.8 ±1.39^{ab}	55.6 ±1.57^{ab}	54.8 ±1.15^{ab}	58.8 ±1.19^{a}
**96**	5 ± 2.55^{c}	46.2 ± 4.51^{b}	62.2 ±2.87^{a}	59.2 ±2.7^{a}	63.2 ±1.59^{a}	60.2 ±1.27^{a}	59.6 ±1.09^{a}	65.2 ±1.16^{a}
	**PD**	**E 17.5**	**AS 2.5**	**AA 2.5**	**CS 2.5**	**CP 2.5**	**SH 2.5**	**CO 2.5**
**24**	6 ± 3.39^{c}	21.8 ±4.22^{bc}	34.6 ±3.07^{ab}	34.6 ±2.11^{a}	36.8 ±1.77^{a}	34 ±1.91^{a}	33.2 ±1.32^{ab}	36.6 ±1.59^{a}
**48**	5.8 ± 1.3^{c}	30.6 ± 3.92^{b}	41.2 ±3.73^{ab}	47.2 ±2.88^{a}	43.8 ±2.44^{ab}	39.6 ±1.77^{ab}	40 ±1.53^{ab}	43.2±2.11^{ab}
**72**	5.8 ±1.48^{c}	30.2 ± 3.05^{b}	48.4 ±3.33^{a}	52.8 ±2.3^{a}	48.2 ±2.67^{a}	45.8 ±0.93^{a}	46.2 ±1.35^{a}	51 ±2.34^{a}
**96**	5 ± 2.55^{c}	34.8 ± 4.01^{b}	52.4 ±2.03^{a}	57.2 ±1.82^{a}	53.4 ±1.44^{a}	50.8 ±1.06^{a}	50.8 ±1.27^{a}	56.2 ±1.18^{a}
	**PD**	**E 8.75**	**AS 1.25**	**AA 1.25**	**CS 1.25**	**CP 1.25**	**SH 1.25**	**CO 1.25**
**24**	6 ± 3.39^{b}	20.8 ±2.62^{ab}	26 ±3.98^{a}	28.4 ±1.68^{a}	28.6 ±2.2^{a}	25 ±2.24^{a}	24.6 ±1.92^{a}	27.8 ±1.86^{a}
**48**	5.8 ± 1.3^{b}	28.8 ±4.45^{ab}	33.2 ±4.39^{a}	34.6 ±3.98^{a}	35.4 ±2.23^{a}	32.2 ±2.6^{a}	32 ±2.34^{a}	35.4 ±1.76^{a}
**72**	5.8 ±1.48^{b}	30.4 ± 4.73^{a}	38.8 ±3.94^{a}	39 ±3.38^{a}	39.2 ±2.22^{a}	36.2 ±1.94^{a}	35.2 ±1.76^{a}	41 ±2.49^{a}
**96**	5 ± 2.55^{b}	35.6 ± 4.11^{a}	43 ±3.58^{a}	43 ±3.56^{a}	45.4 ±1.69^{a}	42.6 ±1.21^{a}	42.4 ±1.21^{a}	46.8 ±2.18^{a}
	**PD**	**E 4.375**	**AS 0.625**	**AA 0.625**	**CS 0.625**	**CP 0.625**	**SH 0.625**	**CO 0.625**
**24**	6 ± 3.39^{b}	17.6 ±3.27^{ab}	21.6 ±2.79^{a}	24.6 ±2.19^{a}	25 ±1.38^{a}	20.8 ±1.4^{a}	20.4 ±1.09^{a}	24 ±1.2^{a}
**48**	5.8 ± 1.3^{b}	23 ± 1.93^{a}	25.6 ± 1.56^{a}	33 ± 1.8^{a}	31.2 ±1.8^{a}	26.4 ± 1.57^{a}	26 ± 1.25^{a}	30.6 ±1.63^{a}
**72**	5.8 ±1.48^{c}	25.4 ±3.63^{ab}	28.8 ± 1.8^{ab}	36.2 ± 1.64^{b}	29.8 ± 1.28^{ab}	26.8 ± 1.57^{a}	28.8 ± 2.35^{ab}	32 ± 1.32^{ab}
**96**	5 ± 2.55^{c}	28 ± 3.08^{b}	31 ± 2.27^{ab}	38.8 ± 1.51^{a}	32.4 ±1.81^{ab}	28.8 ± 1.81^{b}	29.6 ± 0.91^{b}	33.6±1.22^{ab}
	**PD**	**E 2.187**	**AS 0.312**	**AA 0.312**	**CS 0.312**	**CP 0.312**	**SH 0.312**	**CO 0.312**
**24**	6 ± 3.39^{b}	14.4 ±2.53^{ab}	16.6 ± 2.6^{ab}	24 ± 3.06^{a}	19 ±1.05^{ab}	15.6 ± 1.25^{b}	15.8 ± 1.24^{b}	18.6±1.24^{ab}
**48**	5.8 ± 1.3^{c}	17.8 ±1.36^{bc}	20.8 ± 3.2^{ab}	30 ± 2.94^{a}	23.2 ±1.59^{ab}	19.4 ± 1.4^{bc}	19.6 ± 1.38^{bc}	23.2±1.18^{ab}
**72**	5.8 ±1.48^{c}	20.6 ±2.38^{bc}	23.4 ± 2.7^{bc}	32.4 ± 2.73^{b}	22.2 ±1.56^{bc}	21.2 ± 1.2^{ac}	21.2 ± 1.57^{ac}	26 ± 2.51^{ab}
**96**	5 ± 2.55^{c}	21.2 ± 1.76^{b}	25.2 ± 2.49^{b}	34.8 ± 2.63^{a}	22.8 ±1.07^{b}	23 ± 1.82^{b}	22 ± 0.89^{b}	25.8 ±1.33^{b}

SDM-standard deviation of mean, E (ethanol), PD (potassium dichromate), AS (*A. sativum*), AA (*A. absinthium*), CP (*C. pepo*), CS (*C. sativum*), SH (*S. hortensis*), CO (*C. officinalis*). Values with no common superscript in a column within an experiment were significantly different ($p \leq 0.05$).
